# Prognostic indicators for perioperative survival after diaphragmatic herniorrhaphy in cats and dogs: 96 cases (2001-2013)

**DOI:** 10.1186/s12917-016-0926-y

**Published:** 2017-01-07

**Authors:** Claire Legallet, Kelley Thieman Mankin, Laura E. Selmic

**Affiliations:** 1 Department of Small Animal Clinical Sciences (Thieman Mankin, Legallet), College of Veterinary Medicine, Texas A&M University, College Station, TX 77843-4474 USA; 2 The Department of Veterinary Clinical Medicine (Selmic), College of Veterinary Medicine, University of Illinois at Urbana-Champaign, Urbana, IL 61802 USA

**Keywords:** Cat, Diaphragmatic herniorrhaphy, Dog, Surgery, Trauma

## Abstract

**Background:**

To determine associations between perioperative mortality after surgery for traumatic diaphragmatic hernia, medical records of 17 cats and 79 dogs that underwent diaphragmatic herniorrhaphy were reviewed.

**Results:**

The combined perioperative survival rate was 81.3% (88.2% in cats and 79.8% in dogs). Data from acute and chronic cases was assessed separately. Of the acute cases (12 cats and 48 dogs), 10 cats (83.3%) and 38 dogs (79.2%) survived to discharge. Of the chronic cases (5 cats and 31 dogs), 5 cats (100%) and 25 dogs (80.6%) survived to discharge. The time between trauma and surgery, trauma and admission, and admission and surgery were not associated with survival. For cats and dogs, increased duration of anesthesia and surgical procedure were associated with increased mortality (*P* = 0.0013 and 0.004, respectively). Animals with concurrent soft tissue injuries had a 4.3 times greater odds of mortality than those without soft tissue injury (*P* = 0.01). Animals with concurrent soft tissue and orthopedic injuries had a 7.3 times greater odds of mortality than those without soft tissue and orthopedic injuries (*P* = 0.004). Animals that were oxygen dependent had a 5.0 times greater odds of mortality than those that were not (*P* = 0.02). No other variables were significantly associated with survival.

**Conclusions:**

For cats and dogs that underwent surgery for traumatic diaphragmatic hernia, increased anesthetic duration, increased duration of surgical procedure, concurrent soft tissue injuries, concurrent soft tissue and orthopedic injuries, and perioperative oxygen dependence were associated with increased mortality.

## Background

Diaphragmatic hernia is a common injury occurring in cats and dogs. Trauma caused by motor vehicle injury is the most common cause of diaphragmatic hernia and leads to a variety of clinical signs, with the most common being respiratory difficulty [1–12]. Following surgical treatment, the reported survival rate is 54–90% [1, 3, 5, 7–11, 13].

Multiple factors have been reported to influence the rate of survival, including the timing of surgical intervention [1, 2, 6–8, 10, 11]. In one study, surgical intervention within 24 h of trauma, or more than 1 year after trauma resulted in significantly higher mortality rates in dogs [1]. However, the aforementioned study was flawed in design and power [1]. Dogs with acute and chronic herniation, and congenital and traumatic herniation were analysed together [1]. Additionally, the authors of this study report that the 62.5% chronic herniation mortality rate was falsely increased by including dogs that died of unrelated medical problems [1]. Further, only 8 dogs underwent surgery over a year following trauma for chronic hernia repair. Therefore, conclusions drawn from this data should be viewed with suspicion. 40 dogs underwent surgery within 24 h of trauma. Although stabilization procedures were not discussed, the primary cause of death was listed as “shock”. As a result of this previously published study, some investigators recommend delaying surgical intervention for a minimum of 24 h to permit stabilization of the patient prior to surgery [1, 7]. Stabilizing animals prior to anesthesia and surgery may reduce the mortality rates due to complications from dehydration, hypovolemic and distributive shock, and hypoxemia [11]. However, a more recent study has shown no significant impact of early surgical intervention on perioperative mortality rate [8]. This study evaluated 92 dogs and cats undergoing diaphragmatic herniorrhaphy for traumatic herniation [8]. Animals with acute and chronic herniation were evaluated separately [8]. In animals with acute herniation, this study found no associations between perioperative survival and time from trauma to admission, time between admission and surgery, or time from trauma to surgery [8]. Contrary to the previous study, this study suggests early intervention is not associated with poor survival outcomes [8].

The purpose of this retrospective study was to examine factors influencing survival in dogs and cats undergoing diaphragmatic herniorrhaphy.

## Methods

### Criteria for selection of cases

An electronic medical record search was performed to identify cats and dogs undergoing diaphragmatic herniorrhaphy as treatment for traumatic diaphragmatic hernia at Texas A&M Veterinary Teaching Hospital between 1^st^ October 2001 and 31^st^ April 2014. Criterion for inclusion was surgical treatment of a traumatic diaphragmatic hernia. Diagnosis of diaphragmatic hernia was made by use of radiography and/or ultrasonography, and confirmed by surgical exploration. In order to determine if the diaphragmatic hernia was traumatic or congenital, the medical record was reviewed and a combination of history, location of hernia, concurrent injuries detected, and surgical findings (presence of adhesions) were used.

### Procedures

The following information was obtained from medical records: age, sex and neuter status, and body weight; respiratory rate, pulse oximetry (SpO_2_), and blood lactate at presentation; cause of diaphragmatic hernia (if known); concurrent soft tissue and/or orthopedic injuries; times from trauma to admission (TA), trauma to surgery (TS), and admission to surgery (AS); duration of anaesthesia and surgery; organs herniated; additional surgical procedures performed during herniorrhaphy; intraoperative and postoperative complications; times from admission to discharge, and surgery to discharge. TA time was based on information provided by the owner or referring veterinarian. For animals without known trauma, TA was calculated from the onset of symptoms. Acute and chronic diaphragmatic hernias were defined as TA periods ≤ 14 days and > 14 days, respectively [8, 10]. Respiratory distress was considered to be present if “dyspnea” or “respiratory distress” were recorded in the medical record [8]. Animals were also evaluated for respiratory distress based on respiratory rate and SpO_2_ at admission: Animals with respiratory rates > 40 breaths per minute and/or SpO_2_ < 95% [14] were defined as being in respiratory distress. Animals were classified into survival groups as alive to discharge from hospital or death prior to discharge from hospital, including animals euthanized during or after surgery.

### Treatment

All animals underwent general anesthesia, manual ventilation and diaphragmatic herniorrhaphy by standard ventral midline abdominal approach.

### Pre- and postoperative care

After surgery, all of the animals were recovered in the intensive care unit (ICU). All of the animals were closely monitored while in the ICU. All animals had their respiratory rate monitored at least every 2 h. All animals were treated with an opiate. The opiate was administered on presentation, as a premedication and/or induction agent, and intra- or postoperatively. Different types of opiates and a range of doses were used based on clinician preference. Many animals also received a non-steroidal anti-inflammatory drug (NSAID), while some received steroids. Many animals underwent electrolyte monitoring and received fluids and additional supportive care as clinically indicated.

### Statistical analyses

Continuous variables were tested for normality using histograms, skewness, kurtosis, and Shapiro-Wilk tests. If the variables were normally distributed, the mean and standard deviations were presented. For non-normally distributed variables, the median and range were presented. Categorical variables were presented by frequency and percentages.

Kaplan-Meier methodology was used to calculate the median and 95% confidence interval for time from TA, TS, AS, anesthetic duration, surgery duration, and admission to discharge or death for all cases and stratified by species, duration of hernia (acute vs. chronic and for whether animals survived or died in the perioperative period. Log-rank tests were used to assess for differences in these time-to-event variables for species, duration of hernia and for animals that survived to discharge and those that did not survive.

Univariable logistic regression analysis was used to test for associations between mortality and patient demographics (sex and neuter status, age, weight, and species), characteristics at presentation (duration of diaphragmatic hernia, presence of dyspnea, tachypnea >40bpm, pulse oximetry <95%, necessity of thoracocentesis, elevation of serum lactate, number of injuries, presence of concurrent orthopedic injuries, and orthopedic and soft tissue injuries). In addition, associations between different operative factors (time < 24 h following admission, number of anesthetic procedures or number of surgical procedures, number of organs herniated, and which organs were herniated) were tested. A logistic regression model was used with variables of duration or hernia and whether surgery was performed within 24 h of trauma to evaluate effect of these variables on mortality given the findings of previous studies. Multivariable logistic regression analysis was not performed given the low number of animals that died (18). Odds ratios (OR) were calculated with 95% Wald confidence intervals (CI) for each variable.

Statistical significance was set at *p* < 0.05. Statistical analyses were performed using commercially available software^1^.

## Results

Ninety-six animals, 17 cats and 79 dogs, were included in the study. The most common cause of diaphragmatic hernia was motor vehicle accident (Table 1).

**Table 1:** Cause of diaphragmatic hernia in cats and dogs that underwent diaphragmatic herniorrhaphy

Cause	Cats *n* = 17	Dogs *n* = 79	All animals (*n* = 96)
Motor vehicle accident	5 (29.4%)	39 (49.4%)	44 (45.8%)
Unknown/suspected trauma	9 (52.9%)	33 (41.8%)	42 (43.8%)
Other trauma	3 (17.6%)	7 (8.9%)	10 (10.4%)

Seventeen cats were included in the study. Breeds included domestic mix breed cat (*n* = 16, 94.1%) and Rag Doll (*n* = 1, 5.9%). Sex, neuter status, age, and weight were not associated with mortality (Table 2).

**Table 2:** Patient characteristics of cats and dogs that underwent diaphragmatic herniorrhaphy and associations with mortality

Characteristic	Category	Dogs (*n* = 79)	Cats (*n* = 17)	All cases (*n* = 96)	OR (95% CI)	*P*-value
Species	Cat Dog	–	–	–	Ref 1.9 (0.4–9.2)	0.42
Sex and neuter status	Female spayed Female intact Male castrated Male intact	31 (39.2%) 9 (11.4%) 20 (25.3%) 19 (24.1%)	4 (23.5%) 1 (5.9%) 12 (70.6%) 0 (0.0%)	35 (36.5%) 10 (10.4%) 32 (33.3%) 19 (19.8%)	0.8 (0–6.6) 2.9 (0.5–30.7) 2.3 (0.4–25.8) Ref	0.84 0.33 0.53 –
Sex	Female Male	40 (50.6%) 39 (49.4%)	5 (29.4%) 12 (70.6%)	45 (46.9%) 51 (53.1%)	–	–
Age at diagnosis (yr)	Median, range	3.0 (0.2–12.9)	4.7, 1.5–8.5	3.1, 0.2–12.9	1.1 (1.0–1.3)	0.10
Weight (kg)	Median, range	10.6, 2.2–52.0	4.6, 2.3–8.0	8.0, 2.2–52.0	1.0 (1.0–1.1)	0.33

Results are for univariable logistic regression analysis. Values were considered significant at *p* < 0.05. *OR* Odds ratio, *CI* Confidence interval, *Ref* Reference category, *yr* years, *kg* kilograms

Two cats with acute herniation died. Both of the cats that died had concurrent injuries, which were treated surgically. One cat underwent nephrectomy due to an avulsed renal vein, artery and ureter. This cat underwent cardiopulmonary arrest once prior to celiotomy and was successfully resuscitated, but arrested again intraoperatively and died. The other cat that died suffered a penetrating thoracic wound and multiple abdominal punctures after being attacked by a dog. The cat underwent celiotomy and thoracotomy, diaphragmatic herniorrhaphy, two partial lung lobectomies, and repair of the thoracic and abdominal wounds, but became septic and was euthanized 4 days postoperatively.

Seventy-nine dogs were included in the study. The commonly represented breeds were mixed breed (10, 12.7%), Labrador retriever (9, 11.4%), and Chihuahua (5, 6.3%). Other breeds included Alaskan Malamute, Australian Shepherd, Basset Hound, Beagle, Black Mouth Cur, Bloodhound, Border Collie, Boston Terrier, Boxer, Cairn Terrier, Cardigan Welsh Corgi, Catahoula Hog Dog, Cocker Spaniel, Coton de Tulear, English Bulldog, Fox Terrier, German Shepherd, Golden Retriever, Great Pyrenees, Italian Greyhound, Jack Russell Terrier, Maltese, Miniature Dachshund, Miniature Pinscher, Miniature Poodle, Pembroke Welsh Corgi, Pomeranian, Rat Terrier, Shetland Sheepdog, Shih Tzu, Standard Dachshund, Standard Poodle, Treeing Walker Coon Hound, Weimaraner and West Highland Terrier. Sex, neuter status, age, and weight were not associated with mortality (Table 2).

For all animals, oxygen dependence at any time during hospitalization was associated with increased mortality (Table 3). Oxygen dependence preoperatively and postoperatively were associated with increased mortality (Table 3). However, respiratory rate, presence of dyspnoea, pulse oximetry, thoracocentesis, serum lactate levels at presentation, and hernia duration were not associated with mortality (Table 3).

**Table 3:** Presenting characteristics of cats and dogs that underwent diaphragmatic herniorrhaphy and associations with mortality

Characteristic	Category	Dogs (*n* = 79)	Cats (*n* = 17)	All cases (*n* = 96)	OR (95% CI)	*P*-value
Hernia duration	Acute Chronic	48 (60.8%) 31 (39.2%)	12 (70.6%) 5 (29.4%)	60 (62.5%) 36 (37.5%)	1.3 (0.4–3.7) Ref	0.69 -
Survival	Acute Chronic All	38/48(79.2%) 25/31(80.6%) 63/79(79.8%)	10/12 (83.3%) 5/5 (100.0%) 15/17(88.2%)	48/60 (80.0%) 30/36 (83.3%) 78/96 (81.3%)	-	–
Respiratory rate at presentation	Mean (SD) >40bpm ≤40bpm	54 (19.0) 55 (73.3%) 20 (26.7%)	52 (17.0) 4 (23.5%) 13 (76.5%)	53 (18.0) 68 (73.9%) 24 (26.1%)	0.3 (0.1–1.0) Ref	0.05 –
Dyspnoea at presentation	Dyspnoeic Non–dyspnoeic	13 (16.5%) 66 (83.5%)	4 (23.5%) 13 (76.5%)	17 (17.7%) 79 (82.3%)	0.9 (0.2–3.6) Ref	0.90
Pulse oximetry at presentation	<95% ≥95%	25 (31.7%) 54 (68.4%)	5 (29.4%) 12 (70.6%)	30 (31.3%) 66 (68.8%)	0.6 (0.2–1.9) Ref	0.36 –
Thoracocentesis at presentation	Performed Not performed	10 (13.2%) 66 (86.8%)	4 (23.5%) 13 (76.5%)	14 (15.0%) 79 (85.0%)	0.8 (0.2–3.9) Ref	0.75
Oxygen dependent	Yes No	46 (58.3%) 33 (41.8%)	9 (53.0%) 8 (47.0%)	55 (57.3%) 41 (42.7%)	5.0 (1.3–18.7) Ref	0.02*
Oxygen dependent	Preoperative Postoperative None	28 (35.4%) 18 (22.8%) 33 (41.8%)	8 (47.1%) 1 (5.9%) 8 (47.1%)	36 (37.5%) 19 (19.8%) 41 (42.7%)	4.2 (1.0–17.1) 5.8 (1.3–26.8) Ref	0.04* 0.02* –
Serum lactate	Elevated Normal Unknown	14 (17.7%) 36 (45.6%) 29 (36.7%)	7 (41.1%) 3 (17.6%) 7 (41.1%)	21 (21.9%) 39 (40.6%) 36 (37.5%)	0.9 (0.2–3.5) Ref –	0.89

Results are for univariable logistic regression analysis

*OR* Odds ratio, *CI* Confidence interval, *Ref* Reference category, *SD* Standard deviation

*Indicates statistically significant difference

For all animals, presence of concurrent soft tissue injuries were associated with increased mortality (Table 4). Additionally, presence of concurrent orthopedic and soft tissue injuries were associated with increased mortality (Table 4). However, concurrent orthopedic injuries, number of injuries, number of surgeries, number of anesthetic episodes, number of organs herniated and organs herniated had no association with mortality (Table 4). The most common concurrent surgery performed with diaphragmatic herniorrhaphy was amputation in cats and ovariohysterectomy or orchiectomy in dogs (Table 5).

**Table 4:** Anesthesia and operative details of diaphragmatic herniorrhaphy in cats and dogs and associations with mortality

Characteristic	Category	Dogs (*n* = 79)	Cats (*n* = 17)	All cases (*n* = 96)	OR (95% CI)	*P* value
Anesthetic episodes during hospitalization	Median (range)	1 (0–2)	1 (1)	1.0 (0–2)	0.2 (0.0–1.8)	0.15
Concurrent surgeries	Median (range)	1 (1–4)	1 (1–3)	1 (1–4)	1.8 (0.9–3.7)	0.09
Number of injuries	Median (range)	1 (1–5)	2 (1–4)	1 (1–5)	1.5 (1.0–2.4)	0.05
Concurrent orthopedic injuries	Yes No	30 (38.0%) 49 (62.0%)	7 (41.2%) 10 (58.8%)	37 (38.5%) 59 (61.5%)	1.8 (0.6–5.0) Ref	0.27
Concurrent soft tissue injuries	Yes No	11 (13.9%) 68 (86.1%)	6 (35.3%) 11 (64.7%)	17 (17.7%) 79 (82.3%)	4.3 (1.4–13.8) Ref	0.01*
Concurrent orthopedic and soft tissue injuries	Yes No	7 (8.9%) 72 (91.1%)	4 (23.5%) 13 (76.5%)	11 (11.5%) 85 (88.5%)	7.3 (1.9–27.7) Ref	0.004*
Number of organs herniated	Median (range)	3 (0–6)	3 (0–5)	3 (0–6)	0.8 (0.6–1.1)	0.22
Organs herniated	Liver Small intestine Gallbladder Stomach Spleen Omentum Colon Pancreas Kidney Cecum	51 (64.6%) 43 (54.4%) 40 (50.6%) 34 (43.0%) 31 (39.2%) 17 (21.5%) 8 (10.1%) 3 (3.8%) 4 (5.1%) 3 (3.8%)	9 (52.9%) 8 (47.1%) 6 (37.5%) 9 (52.9%) 7 (41.2%) 1 (5.9%) 1 (5.9%) 1 (5.9%) 2 (12.5%) 0 (0.0%)	60 (62.5%) 51 (53.1%) 46 (48.4%) 43 (44.8%) 38 (39.6%) 18 (19.0%) 9 (9.4%) 4 (4.2%) 6 (6.3%) 3 (3.2%)	0.5 (0.2–1.5) 0.9 (0.3–2.4) 0.5 (0.2–1.4) 0.6 (0.2–1.6) 0.5 (0.2–1.6) 0.8 (0.2–3.2) 1.3 (0.2–6.7) 4.7 (0.6–35.8) 4.9 (0.9–26.8) –	0.23 0.77 0.16 0.28 0.26 0.78 0.78 0.14 0.06 –

Results are for univariable logistic regression analysis

*OR* Odds ratio, *CI* Confidence interval, *Ref* Reference category

*Indicates statistically significant difference

**Table 5:** Concurrent surgical procedures in cats and dogs that underwent surgery for traumatic diaphragmatic herniorrhaphy

Concurrent surgical procedure	Cats 6/17	Dogs 28/79
Neuter (OHE/castration)	1 (1/0)	9 (5/4)
Resection and anastomosis	0	5
Splenectomy (Partial or complete)	1	4
Wound closure/debridement	1	3
Gastropexy	0	3
Body wall herniorrhaphy	1	2
Intrathoracic surgery (Median sternotomy/Thoracotomy)	0	2 (1/1)
Amputation	2	0
Bladder repair	1	0
Bronchoscopy	0	1
Oesophageal feeding tube placement	1	0
Gastrotomy	0	1
Jejunal feeding tube placement	0	1
Liver lobectomy	0	1
Lung lobectomy	1	0
Nephrectomy	1	1
Prepubic tendon avulsion repair	0	1
PSS ameroid constrictor placement	0	1

*OHE* Ovariohysterectomy, *PSS* Portosystemic shunt. Some animals underwent more than one concurrent surgical procedure

For all animals, increased duration of surgical procedure was associated with increased mortality (Table 6). Additionally, increased anesthetic duration was associated with increased mortality (Table 6). TA, TS, AS, and duration of surgical procedure were not associated with mortality (Table 6). Following adjustment for duration of hernia (acute vs. chronic), there was no association with mortality for patients with TA >24 h vs. ≤ 24 h.

**Table 6:** Diaphragmatic herniorrhaphy surgery and anesthesia timing in cats and dogs and associations with mortality

Characteristic	Category	Animals alive (*n* = 78)	Animals that died (*n* = 18)	All cases (*n* = 96)	*P*-value
Time from trauma to admission (hrs)	Median, 95% CI	102.8, 22.1–178.8	38.6, 2.4–421.9	87.1, 22.1–177.6	0.64
Time from trauma to surgery (hrs)	Median, 95% CI	132.0, 58.6–207.5	47.9, 16.2–429.0	113.8, 48.2–201.0	0.63
Time from admission to surgery (hrs)	Median, 95% CI	16.3, 9.9–21.2	15.2, 7.0–20.1	16.3, 10.3–20.1	0.57
Anaesthesia duration (hrs)	Median, 95% CI	3.0, 2.7–3.2	4.3, 3.2–4.7	3.1, 2.8–3.3	0.0013*
Duration of surgical procedure (hrs)	Median, 95% CI	1.4, 1.3–1.6	1.9, 1.5–2.3	1.53, 1.40–1.67	0.004*

*P*–values reports are the results of the log–rank univariable analysis assessing association with mortality

*Hrs* hours, *CI* Confidence interval

*Indicates statistically significant difference

## Discussion

The perioperative survival rate in the study reported herein was 81.3% overall, with 88.2% of cats and 79.8% of dogs surviving to discharge. The perioperative survival rates following surgical treatment of acute and chronic diaphragmatic herniae was 83.3% and 100% in cats, respectively, and 79.2% and 80.6% in dogs, respectively. These survival rates are consistent with recent reports [1, 3, 5, 7–10, 13]. Chronic diaphragmatic herniae have been associated with a significantly worse prognosis in older reports. The difference in survival rates for chronic diaphragmatic hernia between more recent and older reports may be due to the definition of a chronic diaphragmatic hernia with Gibson et al [8] and Minihan et al [10] defining any hernia treated > 2 weeks after trauma as chronic, while Boudrieau and Muir [1] defined this as > 1 year [1].

In the present study, the mortality rate for cats and dogs was significantly associated with increased duration of surgical procedure, increased anesthetic duration, concurrent soft tissue injuries, concurrent soft tissue and orthopedic injuries, and perioperative oxygen dependence. Animals with increased duration of surgical procedure or anesthetic duration had an increase in mortality. It is possible that increased duration of surgical procedure and anesthetic duration themselves actually lead to an increase in mortality. However, we suspect that animals with more severe injuries and additional intrathoracic trauma may have been slower to recover from anesthesia, and therefore had longer anesthetic times and increased mortality. It is also possible that more severe trauma lead to more significant diaphragmatic disruption and resultant difficulty performing the herniorrhaphy, and therefore longer surgery and anesthetic times. Alternatively, extended anesthetic duration may have been due to comorbidities, unforeseen surgical complications, and/or concurrent surgical procedures. As concurrent surgical procedures increase surgical time and anaesthetic time, this variable was evaluated separately but was not correlated with mortality (*P* = 0.09). However, this may be due to a type II statistical error.

Animals with concurrent orthopedic and soft tissue injuries had a 7.3 times greater odds of mortality than those without orthopedic and soft tissue injuries (Table 4). Additionally, animals with concurrent soft tissue injuries had a 4.3 times greater odds of mortality than those without soft tissue injuries (Table 4). The severity of polytrauma may be associated with the number and severity of injuries and increased mortality rate. However, there was no association with mortality for animals with only diaphragmatic hernia and orthopedic injures.

Animals that were oxygen dependent during hospitalization had a 5.0 times greater odds of mortality than those that were not oxygen dependent (Table 3). Animals that were oxygen dependent were likely to have more severe clinical signs. We suspect that oxygen dependent animals had more significant pulmonary and/or intrathoracic disease than animals that were not oxygen dependent, making it understandable that they were more likely to die during the perioperative period. Animals that were oxygen dependent preoperatively had a 4.2 times greater odds of mortality than those that were not oxygen dependent preoperatively whereas animals that were oxygen dependent postoperatively had a 5.8 times greater odds of mortality than those who were not oxygen dependent postoperatively (Table 3). We do not recommend that oxygen therapy be withheld from animals that require it, but instead the requirement for oxygen supplementation should be recognized as a risk factor for mortality.

Our study did not find an association in perioperative survival rates with timing of surgery. In older reports [1, 7], dogs treated < 24 h after trauma had a significantly increased risk of mortality. However, in the present study and the study by and Gibson et al [8], the timing of surgery did not have a significant impact on survival rates. We expect this finding is due to improvements in critical care pre- and postoperatively, and improvements in anaesthetic management intraoperatively. The original report finding that animals with diaphragmatic hernia have an increased mortality rate when undergoing surgery within 24 h of trauma concluded that herniorrhaphy should be delayed until the animal is stabilized [1]. We refute that the mortality rate is correlated with the timing of surgery.

Many limitations are present in this study due to the retrospective nature. Some medical records were incomplete. Although dyspnea and respiratory distress were qualified with respiratory rate and oxygen saturation, these are subjective assessments. The exact time of trauma was often unclear, and occasionally, no history of a traumatic event was reported by the owner. Therefore, it is possible that a congenital diaphragmatic hernia was mistaken for a traumatic diaphragmatic hernia. While possible, we consider this unlikely. Diaphragmatic hernia was discovered in two dogs after surgical fracture repair, thus prolonging time from admission to surgery and increasing median and maximum time from admission to surgery. There were a low number of cats that underwent diaphragmatic herniorrhaphy, and no associations were made with perioperative survival. Advancements in anesthetic protocol occurred over the 12 years of the study and may have increased perioperative survival. Due to variability of anesthetic protocol and low numbers of cases per year, statistics were not performed to assess the impact of anesthetic protocols. If this study was prospective, a trauma score may have been assessed which may have been correlated with perioperative survival.

## Conclusion

Cats and dogs that underwent longer surgical procedures, underwent longer anesthesia, those with concurrent soft issue injuries, those with concurrent soft tissue and orthopedic injuries, and those that were oxygen dependent during hospitalization had a higher mortality rate. Based on our findings, we do not recommend that every animal with a diaphragmatic hernia be stabilized for 24 h or more prior to surgery. Instead, we recommend that preoperative stabilization be performed, with surgery to follow as indicated clinically.

## Abbreviations

AS: Time from admission to surgery
CI: Confidence intervals
Hr: Hours
Kg: Kilograms
NSAID: Non-steroidal anti-inflammatory drug
OHE: Ovariohysterectomy
OR: Odds ratio
PSS: Portosystemic shunt
Ref: Reference category
SD: Standard deviation
SpO_2_: pulse oximetry
TA: Time from trauma to admission
TS: Time from trauma to surgery
Yr: Years

## Footnotes

1 SAS software, Version 9.3 of the SAS System for PC. Copyright © 2012 SAS Institute Inc. SAS and all other SAS Institute Inc. product or service names are registered trademarks or trademarks of SAS Institute Inc., Cary, NC, USA. ^2^Nova Critical Care Xpress, Nova Biomedical, Waltham, MA. ^3^VITROS 250, Ortho-Clinical Diagnostics, Rochester, NY.
